# Supplementary material for: Optimized and Validated Stability-Indicating RP-HPLC Method for Comprehensive Profiling of Process-Related Impurities and Stress-Induced Degradation Products in Rivaroxaban (XARELTO)®
Source: Int J Mol Sci. 2025 May 15;26(10):4744. doi: 10.3390/ijms26104744 (PMC12112134; doi:10.3390/ijms26104744)
Supplement: Supplementary file 1 [file ijms-26-04744-s001.zip › ijms-3624974-supplementary.pdf]

ORIGINAL RESEARCH
Aktham Mestareehi

Optimized and Validated Stability-Indicating Reverse Phase High Performance Liquid Chromatography Method for Comprehensive Profiling of Process Related Impurities and Stress Induced Degradation Products in Rivaroxaban (XARELTO)

Aktham Mestareehi, PharmD, MS, PhD [1,2,3]*

[1] Department of Applied Pharmaceutical Sciences and Clinical Pharmacy, Faculty of Pharmacy, Isra University, P.O. Box 22, Amman 11622, Jordan

[2] Department of Pharmaceutical Sciences, School of Pharmacy and Health Sciences, Wayne State University, Detroit, MI 48201, USA

[3] Department of Pharmaceutical Sciences, School of Pharmacy, Northeastern Illinois University, Chicago, IL 60625, USA

Correspondence: Aktham Mestareehi, PharmD, MS, PhD*
Detroit, MI, 48201, USA
Email : aktham.mestareehi@med.wayne.edu
https://orcid.org/0000-0002-1478-6310

Solution Preparation Procedures

Preparation of Mobile Phase solutions

Mobile phase A (Buffer pH 2.9): to prepare one liter of 25 mM potassium phosphate monobasic solution at pH 2.90.

Weigh 3.40 grams of Potassium Phosphate Monobasic and transfer it into a 1000 mL beaker. Add 1000 mL of DI water and stir until buffer salt is completely dissolved. Place a calibrated pH probe into the solution and adjust the pH by slowly adding phosphoric acid dropwise. Stop once the desired pH of 2.9 is reached. Filter the buffer by using (0.45µm membrane filter) and sonicate for 20 minutes to remove any air bubbles.

Mobile phase A (Buffer pH 5.0): to prepare one liter of 25 mM sodium acetate solution at pH 5.0

Weigh 2.05 g of Sodium Acetate and transfer it into a 1000 mL beaker. Add 1000 mL of DI water and stir until buffer salt is completely dissolved. Place a calibrated pH probe into the solution and adjust the pH by slowly adding glacial acetic acid dropwise. Stop once the desired pH of 5.0 is reached. Filter the buffer by using (0.45µm membrane filter) and sonicate for 20 minutes to remove any air bubbles.

Mobile phase A (Buffer pH 7.0): to prepare one liter of 25 mM potassium phosphate dibasic solution at pH 7.0

Weigh 4.36 g of Potassium Phosphate Dibasic and transfer it into a 1000 mL beaker. Add 1000 mL of DI water and stir until buffer salt is completely dissolved. Place a calibrated pH probe into the solution and adjust the pH by slowly adding phosphoric acid dropwise. Stop once the desired pH of 7.0 is reached. Filter the buffer by using (0.45µm membrane filter) and sonicate for 20 minutes to remove any air bubbles.

Mobile phase B (100% Acetonitrile)

Transfer 1000 mL of ACN into the mobile phase reservoir and sonicate for 20 minutes to remove air bubbles.

Stock solution of Rivaroxaban (10,000 ppm)

Weigh 500 mg of Rivaroxaban and transfer it into a 50 mL volumetric flask. Add 25 mL of ACN: DI water (70:30 v/v) and sonicate for 20 minutes or until Rivaroxaban is completely dissolved. Complete the volume to the mark with ACN: DI water (70:30 v/v) and shake it thoroughly.

Stock solution of Rivaroxaban (1,000 ppm)

Transfer 5.0 mL of stock solution Rivaroxaban (10,000ppm) into a 50 ml volumetric flask. Complete the volume to the mark with ACN: DI water (70:30 v/v) and shake it thoroughly.

Solution Preparation for Forced Degradation Study

Stock solution of Rivaroxaban (3500 ppm)

Weigh 350 mg of Rivaroxaban and transfer it into a 100 mL volumetric flask. Add 25 mL of ACN: DI water (70:30 v/v) and sonicate for 20 minutes or until Rivaroxaban is completely dissolved. Complete the volume to the mark with ACN: DI water (70:30 v/v) and shake it thoroughly.

Stock solution of 6N HCl: Transfer 49.6 mL of concentrated 12.1N HCl into a 100 mL volumetric flask. Complete the volume to the mark with DI water and shake it thoroughly.

Stock solution of 3N HCl: Transfer 50 mL of concentrated 6 N HCl into a 100 mL volumetric flask. Complete the volume to the mark with DI water and shake it thoroughly.

Stock solution of 1N HCl: Transfer 33.4 mL of 3N HCl solution into a 100 mL volumetric flask. Complete the volume to the mark with DI water and shake it thoroughly.

Stock solution of 0.5N HCl: Transfer 25 mL of 1N HCl solution into a 50 mL volumetric flask. Complete the volume to the mark with DI water and shake it thoroughly.

Stock solution of 0.1N HCl: Transfer 5 mL of 1N HCl solution into a 50 mL volumetric flask. Complete the volume to the mark with DI water and shake it thoroughly.

Stock solution of 0.05N HCl: Transfer 2.5 mL of 1N HCl solution into a 50 mL volumetric flask. Complete the volume to the mark with DI water and shake it thoroughly.

Stock solution of 0.01N HCl: Transfer 5 mL of 0.1N HCl solution into a 50 mL volumetric flask. Complete the volume to the mark with DI water and shake it thoroughly.

Stock solution of 6N NaOH: Weight 24 g of NaOH, dissolve in 80 ml of DI water, transfer it into a 100 ml volumetric flask. Complete the volume to the mark with DI water, and shake it thoroughly.

Stock solution of 3N NaOH: Transfer 50 ml of 6N NaOH solution into a 100 ml volumetric flask. Complete the volume to the mark with DI water and shake it thoroughly.

Stock solution of 1N NaOH: Transfer 16.7 ml of 6N NaOH solution into a 100 ml volumetric flask. Complete the volume to the mark with DI water and shake it thoroughly.

Stock solution of 0.5N NaOH: Transfer 16.7 ml of 3N NaOH solution into a 100 ml volumetric flask. Complete the volume to the mark with DI water and shake it thoroughly.

Stock solution of 0.1N NaOH: Transfer 10 ml of 1N NaOH solution into a 100 ml volumetric flask. Complete the volume to the mark with DI water and shake it thoroughly.

Stock solution of 0.05N NaOH: Transfer 5 ml of 1N NaOH solution into a 100 ml volumetric flask. Complete the volume to the mark with DI water and shake it thoroughly.

Stock solution of 0.01N NaOH: Transfer 10 ml of 0.01N NaOH solution into a 100 ml volumetric flask. Complete the volume to the mark with DI water and shake it thoroughly.

Stock solution of 3% H_2O_2: Transfer 10 ml of 30% H_2O_2 solution into a 100 ml volumetric flask. Complete the volume to the mark with DI water and shake it thoroughly.

Stock solution of 1% H_2O_2: Transfer 33.3 ml of 3% H_2O_2 solution into a 100 ml volumetric flask. Complete the volume to the mark with DI water and shake it thoroughly.

Stock solution of 0.5% H_2O_2: Transfer 16.7 ml of 3% H_2O_2 solution into a 100 ml volumetric flask. Complete the volume to the mark with DI water and shake it thoroughly.

Stock solution of 0.1% H_2O_2: Transfer 10 ml of 1% H_2O_2 solution into a 100 ml volumetric flask. Complete the volume to the mark with DI water and shake it thoroughly.

Stock solution of 0.05% H_2O_2: Transfer 10 ml of 0.5% H_2O_2 solution into a 100 ml volumetric flask. Complete the volume to the mark with DI water and shake it thoroughly.

Samples Solution Preparation for Acid Degradation

Acid stress sample preparation degraded with (6N HCl)

Transfer 2 mL of Rivaroxaban stock solution (3500 ppm) into a screw-cap test tube, add 2 mL of 6 N HCl into it, heat it on a heating block at 75 ºC for 24 hours. The solution was cooled at room temperature and add 2 mL of 6 N NaOH solution to neutralize the acid. The neutralized solution was accurately transferred into a 10 mL volumetric flask, complete the volume to the mark with (30:70 v/v) DI water: ACN, and shake it thoroughly to produce 700 ppm final concentration.

It is very important to check the pH of the solution before injecting into the HPLC system using pH strips to make sure the solution is neutral (pH 7). The solution was filtered with 0.45 μm membrane filter before it was injected into the HPLC system.

Acid stress sample preparation degraded with (3N HCl)

Transfer 2 mL of Rivaroxaban stock solution (3500 ppm) into a screw-cap test tube, add 2 mL of 3 N HCl into it, heat it on a heating block at 75 ºC for 24 hours. The solution was cooled at room temperature, and add 2 mL of 3 N NaOH solution to neutralize the acid. The neutralized solution was accurately transferred into a 10 mL volumetric flask, complete the volume to the mark with (30:70 v/v) DI water: ACN, and shake it thoroughly to produce 700 ppm final concentration.

It is very important to check the pH of the solution before injecting into the HPLC system using pH strips to make sure the solution is neutral (pH 7). The solution was filtered with 0.45 μm membrane filter before it was injected into the HPLC system.

Acid stress sample preparation degraded with (1 N HCl)

Transfer 2 mL of Rivaroxaban stock solution (3500 ppm) into a screw-cap test tube, add 2 mL of 1 N HCl into it, heat it on a heating block at 75 ºC for 24 hours. The solution was cooled at room temperature, and add 2 mL of 1 N NaOH solution to neutralize the acid. The neutralized solution was accurately transferred into a 10 mL volumetric flask, complete the volume to the mark with (30:70 v/v) DI water: ACN, and shake it thoroughly to produce 700 ppm final concentration.

It is very important to check the pH of the solution before injecting into the HPLC system using pH strips in order make sure the solution is neutral (pH 7). The solution was filtered with 0.45 µm membrane filter before it was injected into the HPLC system.

Acid stress sample preparation degraded with (0.5 N HCl)

Transfer 2 mL of Rivaroxaban stock solution (3500 ppm) into a screw-cap test tube, add 2 mL of 0.5 N HCl into it, heat it on a heating block at 75 ºC for 24 hours. The solution was cooled at room temperature, and add 2 mL of 0.5 N NaOH solution to neutralize the acid. The neutralized solution was accurately transferred into a 10 mL volumetric flask, complete the volume to the mark with (30:70 v/v) DI water: ACN, and shake it thoroughly to produce 700 ppm final concentration.

It is very important to check the pH of the solution before injecting into the HPLC system using pH strips to make sure the solution is neutral (pH 7). The solution was filtered with 0.45 µm membrane filter before it was injected into the HPLC system.

Acid stress sample preparation degraded with (0.1 N HCl)

Transfer 2 mL of Rivaroxaban stock solution (3500 ppm) into a screw-cap test tube, add 2 mL of 0.1 N HCl into it, heat it on a heating block at 75 ºC for 24 hours. The solution was cooled at room temperature, add 2 mL of 0.1 N NaOH solution to neutralize the acid. The neutralized solution was accurately transferred into a 10 mL volumetric flask, complete the volume to the mark with (30:70 v/v) DI water: ACN, and shake it thoroughly to produce 700 ppm final concentration.

It is very important to check the pH of the solution before injecting into the HPLC system using pH strips to make sure the solution is neutral (pH 7). The solution was filtered with 0.45 µm membrane filter before it was injected into the HPLC system.

Acid stress sample preparation degraded with (0.05 N HCl)

Transfer 2 mL of Rivaroxaban stock solution (3500 ppm) into a screw-cap test tube, add 2 mL of 0.05 N HCl into it, heat it on a heating block at 75 ºC for 24 hours. The solution was cooled at room temperature, add 2 mL of 0.05 N NaOH solution to neutralize the acid. The neutralized solution was

accurately transferred into a 10 mL volumetric flask, complete the volume to the mark with (30:70 v/v) DI water: ACN, and shake it thoroughly to produce 700 ppm final concentration.

It is very important to check the pH of the solution before injecting into the HPLC system using pH strips to make sure the solution is neutral (pH 7). The solution was filtered with 0.45 μm membrane filter before it was injected into the HPLC system.

Sample solutions preparation for base degradation:

<u>Base stress sample preparation degraded with (3N NaOH)</u>

Transfer 2 mL of Rivaroxaban stock solution (3500 ppm) into a screw-cap test tube, add 2 mL of 3N NaOH into it, and heat it on a heating block at 75 º C for 24 hours. The solution was cooled at room temperature, add 2 mL of 3N HCl solution to neutralize the acid. The neutralized solution was accurately transferred into a 10 mL volumetric flask, complete the volume to the mark with (30:70 v/v) DI water: ACN, and shake it thoroughly to produce 700 ppm final concentration.

It is very important to check the pH of the solution before injected into the HPLC system using pH strips in order make sure the solution is neutral (pH 7). The solution was filtered with 0.45 μm membrane filter before it was injected into the HPLC system.

<u>Base stress sample preparation degraded with (1N NaOH)</u>

Transfer 2 mL of Rivaroxaban stock solution (3500 ppm) into a screw-cap test tube, add 2 mL of 1N NaOH into it, heat it on a heating block at 75 ºC for 24 hours. The solution was cooled at room temperature, add 2 mL of 1N HCl solution to neutralize the basic solution. The neutralized solution was accurately transferred into a 10 mL volumetric flask, complete the volume to the mark with (30:70 v/v) DI water: ACN, and shake it thoroughly to produce 700 ppm final concentration.

It is very important to check the pH of the solution before injected into the HPLC system using pH strips in order make sure the solution is neutral (pH 7). The solution was filtered with 0.45 μm membrane filter before it was injected into the HPLC system.

<u>Base stress sample preparation degraded with (0.5N NaOH)</u>

Transfer 2 mL of Rivaroxaban stock solution (3500 ppm) into a screw-cap test tube, add 2 mL of 0.5N NaOH into it, heat it on a heating block at 75 ºC for 24 hours. The solution was cooled at room temperature, add 2 mL of 0.5N HCl solution to neutralize the basic solution. The neutralized solution was accurately transferred into a 10 mL volumetric flask, complete the volume to the mark with (30:70 v/v DI water: ACN, and shake it thoroughly to produce 700 ppm final concentration.

It is very important to check the pH of the solution before injected into the HPLC system using pH strips in order make sure the solution is neutral (pH 7). The solution was filtered with 0.45 µm membrane filter before it was injected into the HPLC system.

<u>Base stress sample preparation degraded with (0.1N NaOH)</u>

Transfer 2 mL of Rivaroxaban stock solution (3500 ppm) into a screw-cap test tube, add 2 mL of 0.1N NaOH into it, heat it on a heating block at 75 ºC for 24 hours. The solution was cooled at room temperature, add 2 mL of 0.1N HCl solution to neutralize the basic solution. The neutralized solution was accurately transferred into a 10 mL volumetric flask, complete the volume to the mark with (30:70 v/v) DI water: ACN, and shake it thoroughly to produce 700 ppm final concentration.

It is very important to check the pH of the solution before injected into the HPLC system using pH strips in order make sure the solution is neutral (pH 7). The solution was filtered with 0.45 µm membrane filter before it was injected into the HPLC system.

<u>Base stress sample preparation degraded with (0.05N NaOH)</u>

Transfer 2 mL of Rivaroxaban stock solution (3500 ppm) into a screw-cap test tube, add 2 mL of 0.05 N NaOH into it, heat it on a heating block at 75 ºC for 24 hours. The solution was cooled at room temperature, add 2 mL of 0.05 N HCl solution to neutralize the basic solution. The neutralized solution was accurately transferred into a 10 mL volumetric flask, complete the volume to the mark with (30:70 v/v) DI water: ACN, and shake it thoroughly to produce 700 ppm final concentration.

It is very important to check the pH of the solution before injected into the HPLC system using pH strips in order make sure the solution is neutral (pH 7). The solution was filtered with 0.45 µm membrane filter before it was injected into the HPLC system.

Sample solutions preparation for oxidation degradation

<u>Oxidation stress sample preparation degraded with (3% H_2O_2)</u>

Transfer 2 mL of Rivaroxaban stock solution (3500 ppm) into a screw-cap test tube, add 2 mL of 3% H_2O_2 into it, heat it on a heating block at 75 ºC for 24 hours. The solution was cooled at room temperature, the solution was accurately transferred into a 10 mL volumetric flask, complete the volume to the mark with (30:70 v/v) DI water: ACN, and shake it thoroughly to produce 700 ppm final concentration. The solution was filtered with 0.45 µm membrane filter before it was injected into the HPLC system.

<u>Oxidation stress sample preparation degraded with (1% H_2O_2)</u>

Transfer 2 mL of Rivaroxaban stock solution (3500 ppm) into a screw-cap test tube, add 2 mL of 1% H_2O_2 into it, heat it on a heating block at 75 ºC for 24 hours. The solution was cooled at room

temperature, the solution was accurately transferred into a 10 mL volumetric flask, complete the volume to the mark with (30:70 v/v) DI water: ACN, and shake it thoroughly to produce 700 ppm final concentration. The solution was filtered with 0.45 µm membrane filter before it was injected into the HPLC system.

Oxidation stress sample preparation degraded with (0.5% H_2O_2)

Transfer 2 mL of Rivaroxaban stock solution (3500 ppm) into a screw-cap test tube, add 2 mL of 0.5% H_2O_2 into it, heat it on a heating block at 75 ºC for 24 hours. The solution was cooled at room temperature, the solution was accurately transferred into a 10 mL volumetric flask, complete the volume to the mark with (30:70 v/v) DI water: ACN, and shake it thoroughly to produce 700 ppm final concentration. The solution was filtered with 0.45 µm membrane filter before it was injected into the HPLC system.

Oxidation stress sample preparation degraded with (0.1% H_2O_2)

Transfer 2 mL of Rivaroxaban stock solution (3500 ppm) into a screw-cap test tube, add 2 mL of 0.1% H_2O_2 into it, heat it on a heating block at 75 ºC for 24 hours. The solution was cooled at room temperature, the solution was accurately transferred into a 10 mL volumetric flask, complete the volume to the mark with (30:70 v/v) DI water: ACN, and shake it thoroughly to produce 700 ppm final concentration. The solution was filtered with 0.45 µm membrane filter before it was injected into the HPLC system.

Solution Preparation for Mix Forced Degradation Study

Stock solution of 0.01N HCl: Transfer 5 mL of 0.1N HCl solution into a 50 mL volumetric flask. Complete the volume to the mark with DI water and shake it thoroughly.

Stock solution of 0.01N NaOH: Transfer 10 ml of 1N NaOH solution into a 100 ml volumetric flask. Complete the volume to the mark with DI water and shake it thoroughly.

Stock solution of 0.05% H2O2: Transfer 10 ml of 0.5% H2O2 solution into a 100 ml volumetric flask. Complete the volume to the mark with DI water and shake it thoroughly.

Acid stress sample preparation degraded with (0.01 N HCl)

Transfer 2 mL of Rivaroxaban stock solution (3500 ppm) into a screw-cap test tube, add 2 mL of 0.01 N HCl into it, heat it on a heating block at 75 ºC for 24 hours. The solution was cooled at room temperature, add 2 mL of 0.01 N NaOH solution to neutralize the acid. The neutralized solution was accurately transferred into a 10 mL volumetric flask, complete the volume to the mark with (30:70 v/v) DI water: ACN, and shake it thoroughly to produce 700 ppm final concentration. It is very important to check the pH of the solution before injecting into the HPLC system using pH strips to make sure the solution is neutral (pH 7). The solution was filtered with 0.45 µm membrane filter before it was injected into the HPLC system.

Base stress sample preparation degraded with (0.01N NaOH)

Transfer 2 mL of Rivaroxaban stock solution (3500 ppm) into a screw-cap test tube, add 2 mL of 0.01N NaOH into it, heat it on a heating block at 75 ºC for 24 hours. The solution was cooled at room temperature, add 2 mL of 0.01N HCl solution to neutralize the basic solution. The neutralized solution was accurately transferred into a 10 mL volumetric flask, complete the volume to the mark with (30:70 v/v) DI water: ACN, and shake it thoroughly to produce 700 ppm final concentration. It is very important to check the pH of the solution before injected into the HPLC system using pH strips in order make sure the solution is neutral (pH 7). The solution was filtered with 0.45 µm membrane filter before it was injected into the HPLC system.

Oxidation stress sample preparation degraded with (0.05% H_2O_2):

Transfer 2 mL of Rivaroxaban stock solution (3500 ppm) into a screw-cap test tube, add 2 mL of 0.05% H_2O_2 into it, heat it on a heating block at 75 ºC for 24 hours. The solution was cooled at room temperature, the solution was accurately transferred into a 10 mL volumetric flask, complete the volume to the mark with (30:70 v/v DI water: ACN, and shake it thoroughly to produce 700 ppm final concentration. The solution was filtered with 0.45 µm membrane filter before it was injected into the HPLC system.

Standard solutions preparation for solution stability

Stock standard solution of Rivaroxaban (10,000 ppm)

Weigh 500 mg of Rivaroxaban and transfer it into a 50 mL volumetric flask. Add 25 mL of ACN: DI water (70:30 v/v) and sonicate for 20 minutes or until Rivaroxaban is completely dissolved. Complete the volume to the mark with ACN: DI water (70:30 v/v) and shake it thoroughly.

Working standard solution of Rivaroxaban (700 ppm)

Transfer 3.5 mL of stock solution Rivaroxaban (10,000ppm) into a 50 ml volumetric flask. Complete the volume to the mark with ACN: DI water (70:30 v/v) and shake it thoroughly.

Standard solutions preparation for linearity study

Stock solution of Rivaroxaban (5,000 ppm)

Weigh 250 mg of Rivaroxaban and transfer it into a 50 mL volumetric flask. Add 25 mL of ACN: DI water (70:30 v/v) and sonicate for 20 minutes or until Rivaroxaban is completely dissolved. Complete the volume to the mark with ACN: DI water (70:30 v/v) and shake it thoroughly.

Stock solution of Rivaroxaban (10 ppm)

Transfer 0.1 mL of stock solution Rivaroxaban (5000ppm) into a 50 mL volumetric flask. Complete the volume to the mark with ACN: DI water (70:30 v/v) and shake it thoroughly.

Solution of Rivaroxaban (2 ppm)

Transfer 5.0 mL of stock solution Rivaroxaban (10 ppm) into a 25 mL volumetric flask. Complete the volume to the mark with ACN: DI water (70:30 v/v) and shake it thoroughly.

Stock solution of Rivaroxaban (1.75 ppm)

Transfer 4.38 mL of stock solution Rivaroxaban (10000ppm) into a 25 mL volumetric flask. Complete the volume to the mark with ACN: DI water (70:30 v/v) and shake it thoroughly.

Stock solution of Rivaroxaban (1.5 ppm)

Transfer 3.75 mL of stock solution Rivaroxaban (10ppm) into a 25 mL volumetric flask. Complete the volume to the mark with ACN: DI water (70:30 v/v) and shake it thoroughly.

Stock solution of Rivaroxaban (1.25 ppm)

Transfer 3.13 mL of stock solution Rivaroxaban (10ppm) into a 25 mL volumetric flask. Complete the volume to the mark with ACN: DI water (70:30 v/v) and shake it thoroughly.

Stock solution of Rivaroxaban (1 ppm)

Transfer 2.5 mL of stock solution Rivaroxaban (10ppm) into a 25 mL volumetric flask. Complete the volume to the mark with ACN: DI water (70:30 v/v) and shake it thoroughly.

Solutions preparation for accuracy study.

Stock solution of Rivaroxaban (5,000 ppm)

Weight 250 mg of Rivaroxaban and transfer it into a 50 mL volumetric flask. Add 25 mL of ACN: DI water (70:30 v/v) and sonicate for 20 minutes or until Rivaroxaban is completely dissolved. Complete the volume to the mark with ACN: DI water (70:30 v/v) and shake it thoroughly.

Stock solution of Rivaroxaban (100 ppm)

Transfer 2.0 mL of stock solution Rivaroxaban (5000ppm) into a 100 mL volumetric flask. Complete the volume to the mark with ACN: DI water (70:30 v/v) and shake it thoroughly.

Stock solution of Rivaroxaban (10 ppm)

Transfer 2.0 mL of stock solution Rivaroxaban (100ppm) into a 100 mL volumetric flask. Complete the volume to the mark with ACN: DI water (70:30 v/v) and shake it thoroughly.

Stock solution of Rivaroxaban (2 ppm)

Transfer 10 mL of stock solution Rivaroxaban (10ppm) into a 50 mL volumetric flask. Complete the volume to the mark with ACN: DI water (70:30 v/v) and shake it thoroughly.

Stock solution of Rivaroxaban (1.5 ppm)

Transfer 7.5 mL of stock solution Rivaroxaban (10ppm) into a 50 mL volumetric flask. Complete the volume to the mark with ACN: DI water (70:30 v/v) and shake it thoroughly.

Stock solution of Rivaroxaban (1 ppm):

Transfer 5.0 mL of stock solution Rivaroxaban (10ppm) into a 50 mL volumetric flask. Complete the volume to the mark with ACN: DI water (70:30 v/v) and shake it thoroughly.

Solutions preparation for method precision study

Stock solution of Rivaroxaban (5,000 ppm)

Weigh 250 mg of Rivaroxaban and transfer it into a 50 mL volumetric flask. Add 25 mL of ACN: DI water (70:30 v/v) and sonicate for 20 minutes or until Rivaroxaban is completely dissolved. Complete the volume to the mark with ACN: DI water (70:30 v/v) and shake it thoroughly.

Stock solution of Rivaroxaban (10 ppm)

Transfer 0.1 mL of stock solution Rivaroxaban (5000ppm) into a 50 mL volumetric flask. Complete the volume to the mark with ACN: DI water (70:30 v/v) and shake it thoroughly.

Stock solution of Rivaroxaban (1.5 ppm)

Transfer 3.75 mL of stock solution Rivaroxaban (10ppm) into a 25 mL volumetric flask. Complete the volume to the mark with ACN: DI water (70:30 v/v) and shake it thoroughly.

Solutions Preparation for Limit of Detection (LOD) Study

Stock solution of Rivaroxaban (5,000 ppm)

Weigh 250 mg of Rivaroxaban and transfer it into a 50 mL volumetric flask. Add 25 mL of ACN: DI water (70:30 v/v) and sonicate for 20 minutes or until Rivaroxaban is completely dissolved. Complete the volume to the mark with ACN: DI water (70:30 v/v) and shake it thoroughly.

Stock solution of Rivaroxaban (10 ppm)

Transfer 0.1 mL of stock solution Rivaroxaban (5000ppm) into a 50 mL volumetric flask. Complete the volume to the mark with ACN: DI water (70:30 v/v) and shake it thoroughly.

Stock solution of Rivaroxaban (5.0 ppm)

Transfer 12.5 mL of stock solution Rivaroxaban (10ppm) into a 25 mL volumetric flask. Complete the volume to the mark with ACN: DI water (70:30 v/v) and shake it thoroughly.

Stock solution of Rivaroxaban (2.0 ppm)

Transfer 5 mL of stock solution Rivaroxaban (10ppm) into a 25 mL volumetric flask. Complete the volume to the mark with ACN: DI water (70:30 v/v) and shake it thoroughly.

Stock solution of Rivaroxaban (1.5 ppm)

Transfer 3.75 mL of stock solution Rivaroxaban (10ppm) into a 25 mL volumetric flask. Complete the volume to the mark with ACN: DI water (70:30 v/v) and shake it thoroughly.

Stock solution of Rivaroxaban (1.0 ppm)

Transfer 2.5 mL of stock solution Rivaroxaban (10ppm) into a 25 mL volumetric flask. Complete the volume to the mark with ACN: DI water (70:30 v/v) and shake it thoroughly.

Stock solution of Rivaroxaban (0.9 ppm)

Transfer 2.25 mL of stock solution Rivaroxaban (10ppm) into a 25 mL volumetric flask. Complete the volume to the mark with ACN: DI water (70:30 v/v) and shake it thoroughly.

Stock solution of Rivaroxaban (0.5 ppm)

Transfer 1.25 mL of stock solution Rivaroxaban (10ppm) into a 25 mL volumetric flask. Complete the volume to the mark with ACN: DI water (70:30 v/v) and shake it thoroughly.

Stock solution of Rivaroxaban (0.4 ppm):

Transfer 1 mL of stock solution Rivaroxaban (10ppm) into a 25 mL volumetric flask. Complete the volume to the mark with ACN: DI water (70:30 v/v) and shake it thoroughly.

Stock solution of Rivaroxaban (0.3 ppm)

Transfer 0.75 mL of stock solution Rivaroxaban (10ppm) into a 25 mL volumetric flask. Complete the volume to the mark with ACN: DI water (70:30 v/v) and shake it thoroughly.

Stock solution of Rivaroxaban (0.2 ppm)

Transfer 0.5 mL of stock solution Rivaroxaban (10ppm) into a 25 mL volumetric flask. Complete the volume to the mark with ACN: DI water (70:30 v/v) and shake it thoroughly.

Supplementary Material Figures

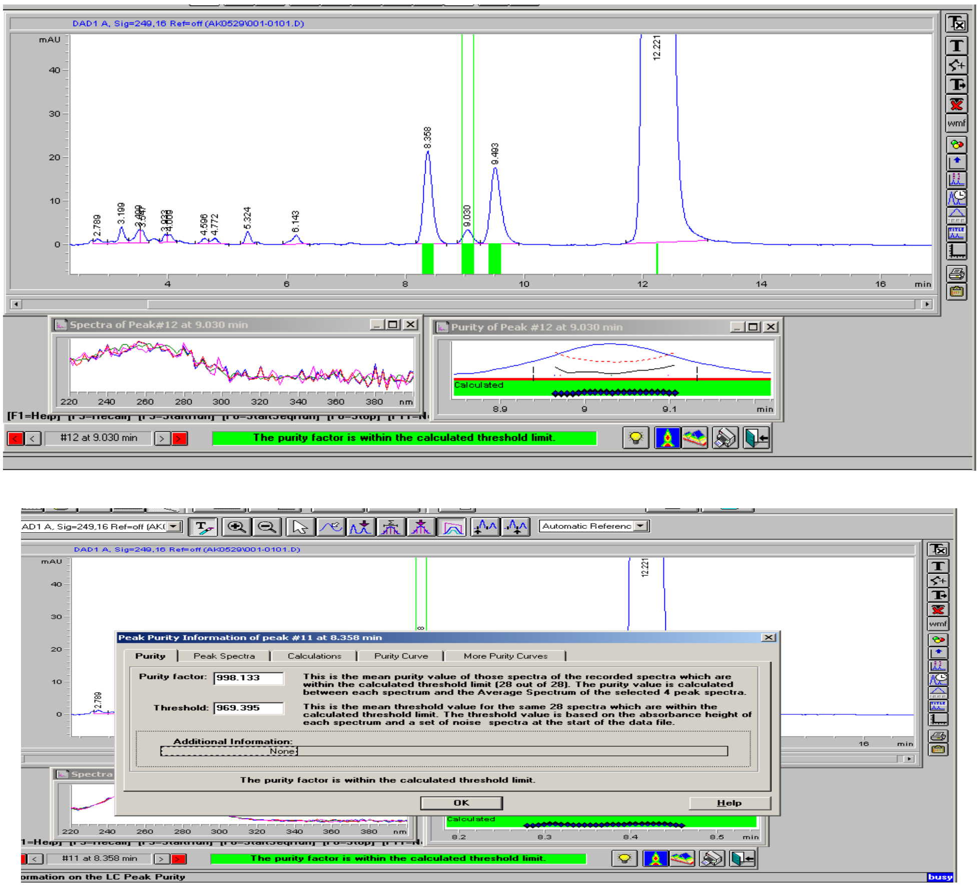

Figure S1. Peak purity of Rivaroxaban degraded to 9.2%

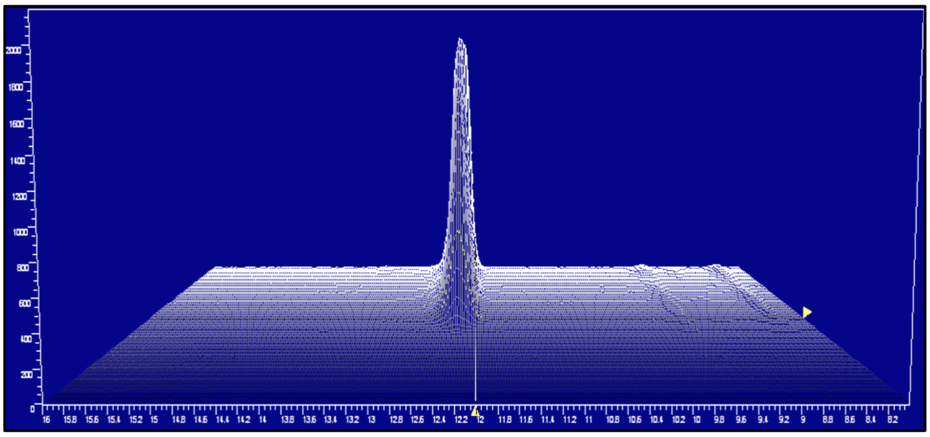

Figure S2. Three-dimension images of Rivaroxaban peak purity.

Chromatographic conditions: Isocratic elution, mobile phase 30:70 ACN/25 mM potassium phosphate buffer monobasic pH 2.9, flow rate 1.0 mL/min, detection wavelength at 249 nm, ambient temperature, 15 µL injection volume, thermo hypersil ODS C_{18} (4.6x250 mm, 5µm) column

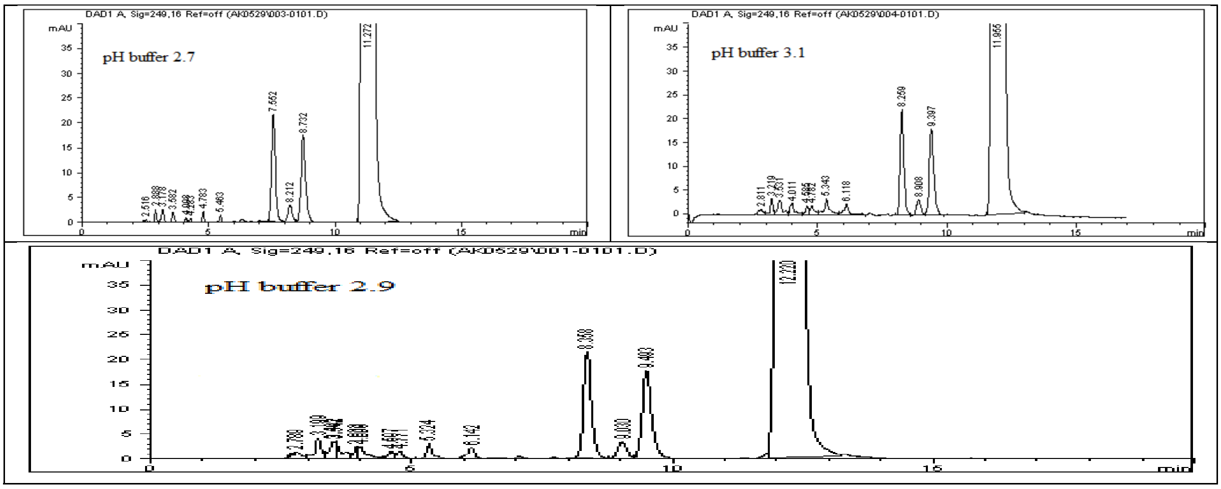

Figure S3: Chromatograms of the mixed degradation sample used in the buffer pH robustness study. Variations were made to the mobile phase buffer pH (2.9 ± 0.2) to evaluate method performance under slight pH fluctuations.

A) Buffer pH: 2.7 B) Buffer pH: 3.1

C) Buffer pH: 2.9 (Developed Method)

Chromatographic conditions: Isocratic elution, mobile phase 30:70 ACN/25 mM potassium phosphate buffer monobasic, flow rate 1.0 mL/min, detection wavelength at 249 nm, ambient temperature, 15 µL injection volume, thermo hypersil ODS C_{18} (4.6x250 mm, 5µm) column.

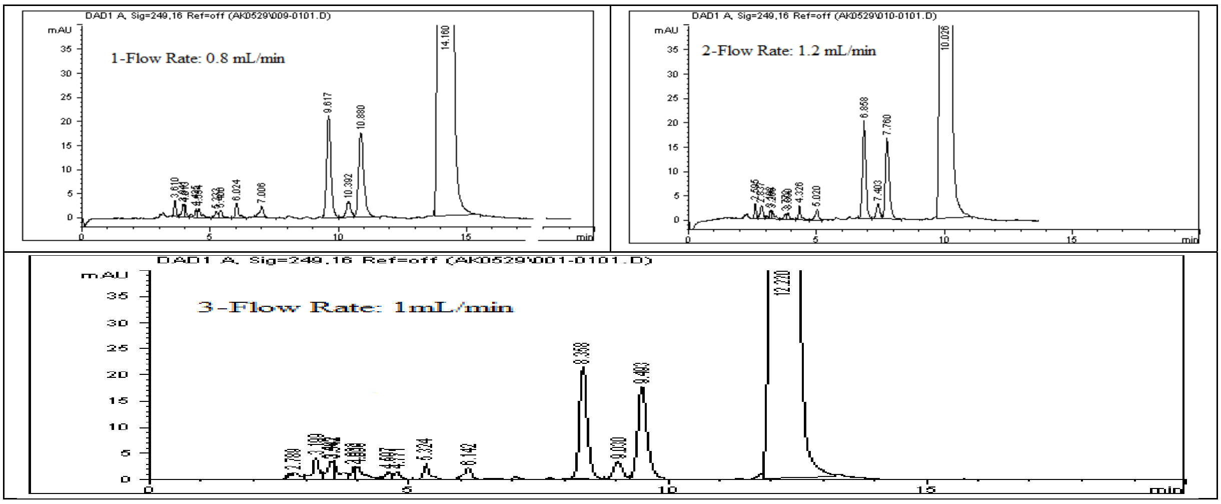

Figure S4. Chromatograms illustrating method robustness with varying flow rates of the mobile phase. The flow rate was adjusted to 1.0 ± 0.2 mL/min to assess the impact on chromatographic performance

1) Flow rate: 0.80 mL/min
2) Flow rate: 1.2 mL/min
3) Flow rate: 1.0 mL/min (Developed Method)

Chromatographic conditions: Isocratic elution, mobile phase 30:70 ACN/25mM potassium phosphate buffer monobasic pH 2.9, detection wavelength at 249 nm, ambient temperature, 15 µL injection volume, thermo hypersil ODS C_{18} (4.6x250 mm, 5µm) column.

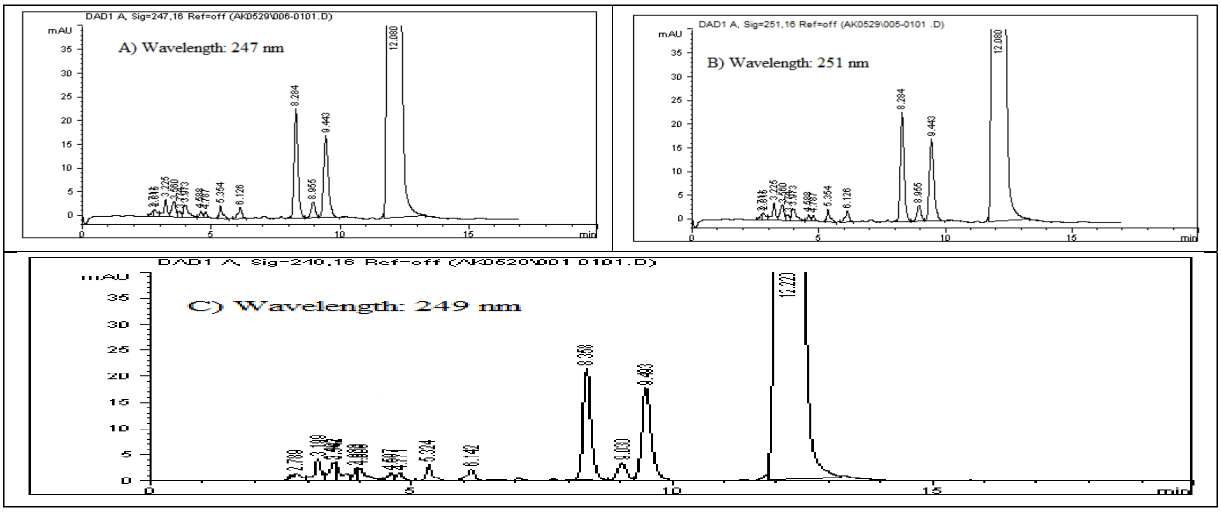

Figure S5. Chromatograms of mixed degradation samples used in the UV wavelength robustness study. The UV detection wavelength was varied at 249 ± 2 nm to evaluate the method's robustness under wavelength fluctuations.

A) Wavelength: 247 nm
B) Wavelength: 251 nm
C) Wavelength: 249 nm (Developed Method)

Chromatographic conditions: Isocratic elution, mobile phase 30:70 ACN/25 mM potassium phosphate buffer monobasic pH 2.9, flow rate 1.0 mL/min, ambient temperature, 15 µL injection volume, thermo hypersil ODS C_{18} (4.6x250 mm, 5µm) column.

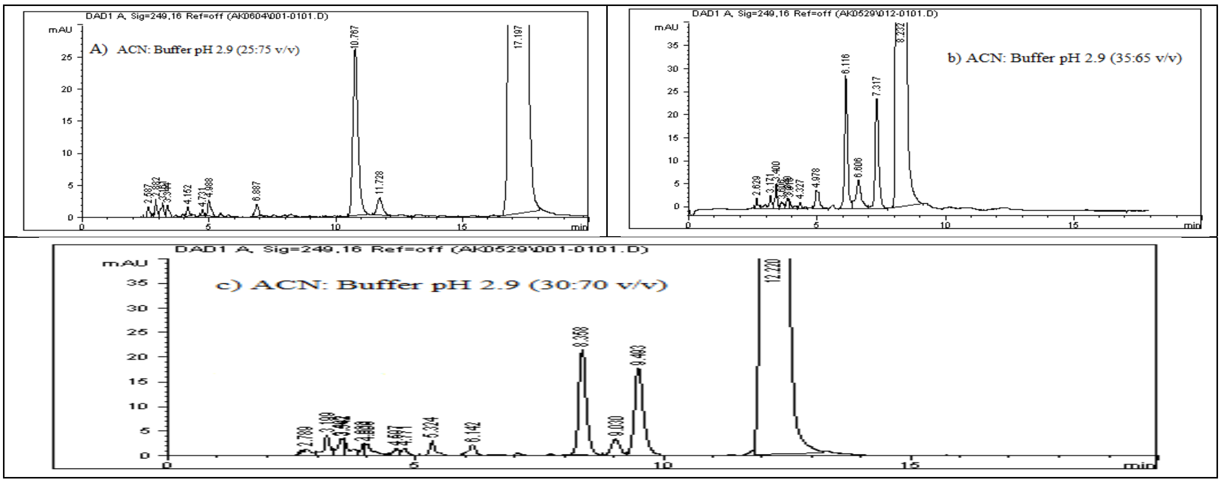

Figure S6. Chromatograms of mixed degradation samples used in the solvent strength robustness study. The isocratic elution composition of acetonitrile to buffer (ACN:Buffer) was varied at 30 ± 5% (v/v) to assess the method's robustness under changes in solvent strength

A) Solvent Strength: 25% ACN
C) Solvent Strength: 30% ACN (Developed Method)
B) Solvent Strength: 35% ACN

Chromatographic conditions: Isocratic elution, mobile phase ACN/25 mM potassium phosphate buffer monobasic pH 2.9, flow rate 1.0 mL/min, detection wavelength at 249 nm, ambient temperature, 15 µL injection volume, thermo hypersil ODS C_{18} (4.6x250 mm, 5µm) column.

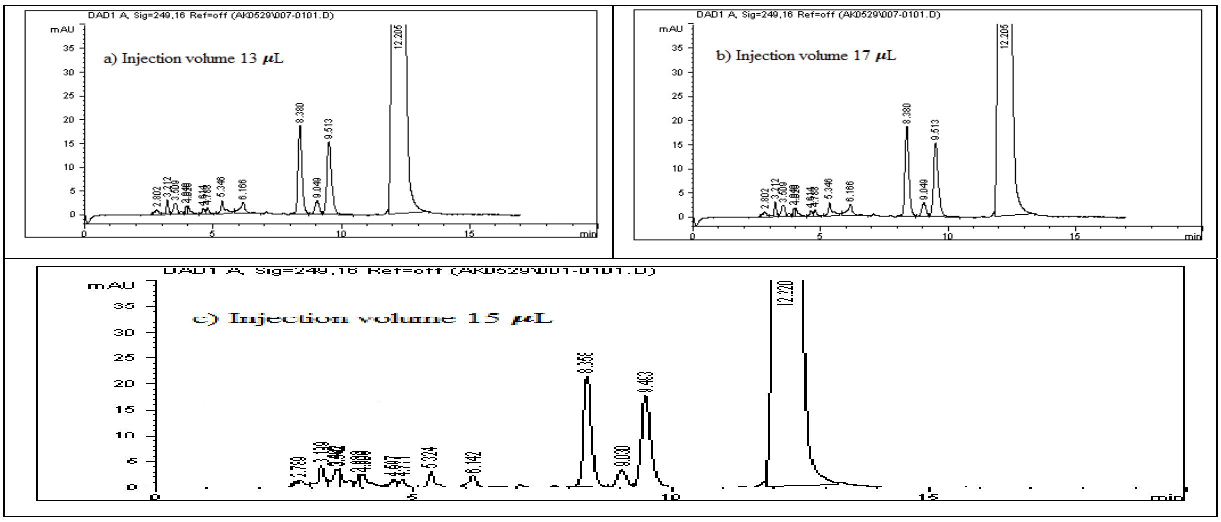

Figure S7. Chromatograms of mixed degradation samples used in the injection volume robustness study. The injection volume was varied at 15 ± 2 µL to evaluate the method's robustness under changes in sample loading

a) Injection volume: 13µL c) Injection volume: 15µL (Developed Method)

b) Injection volume: 17µL

Chromatographic conditions: Isocratic elution, mobile phase 30:70 ACN/25 mM potassium phosphate buffer monobasic pH 2.9, flow rate 1.0 mL/min, detection wavelength at 249 nm, ambient temperature, thermo hypersil ODS C_{18} (4.6x250 mm, 5µm) column.

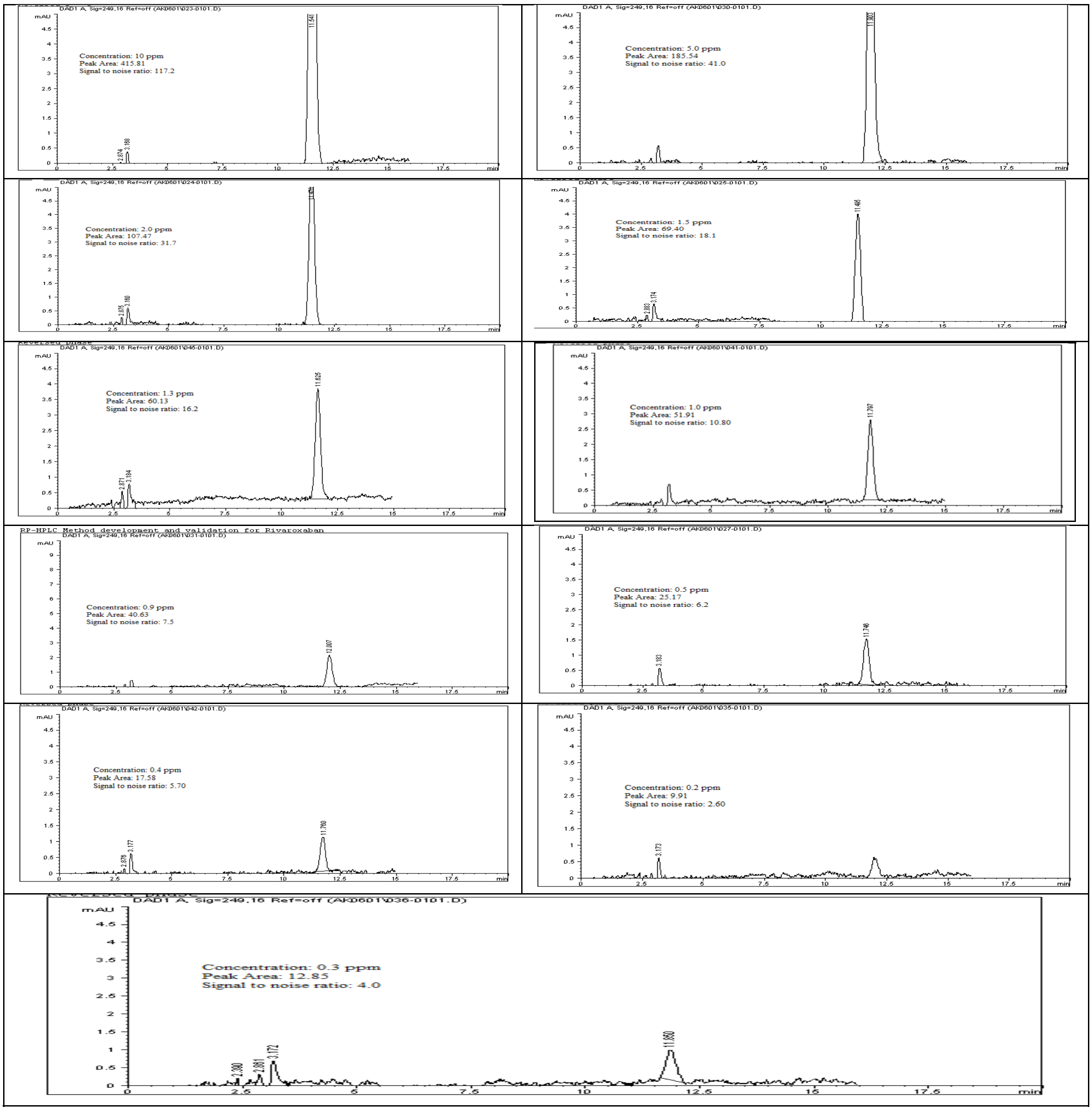

Figure S8. Chromatograms for Limit of detection (LOD)

Chromatographic conditions: Isocratic elution, mobile phase 30:70 ACN/25 mM potassium phosphate buffer monobasic pH 2.9, flow rate 1.0 mL/min, detection wavelength at 249 nm,

ambient temperature, 15 µL injection volume, thermo hypersil ODS C_{18} (4.6x250 mm, 5µm) column.

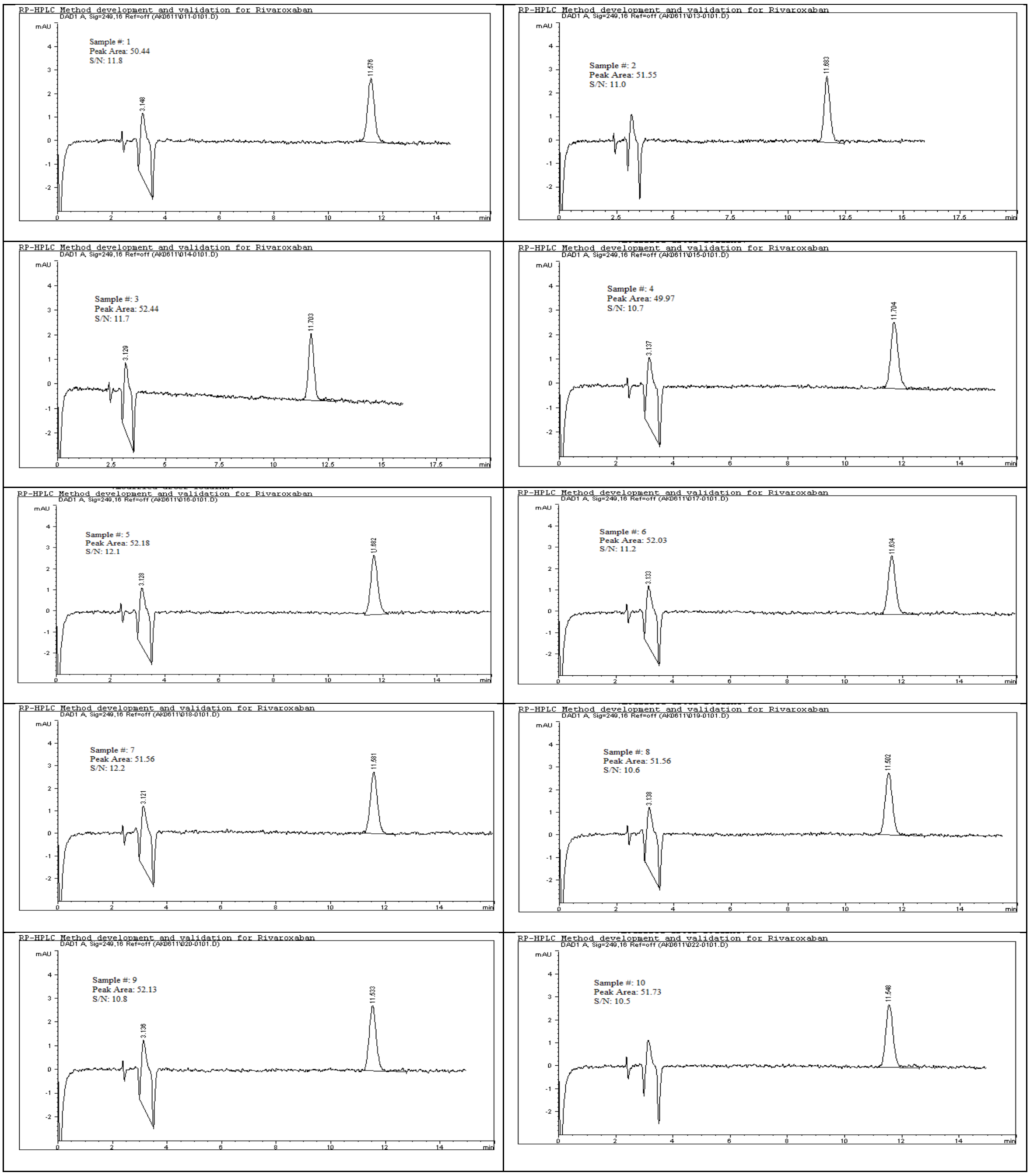

Figure S9. Chromatograms for limit of quantitation (LOQ) study for 1.0 ppm

Chromatographic conditions: Isocratic elution, mobile phase 30:70 ACN/25 mM potassium phosphate buffer monobasic pH 2.9, flow rate 1.0 mL/min, detection wavelength at 249 nm, ambient temperature, 15 µL injection volume, thermo hypersil ODS C_{18} (4.6x250 mm, 5µm) column.

Supplementary Material Tables

Table S1. Mixed Degradation Study Results Under Optimized Chromatographic Conditions

Stress condition	Exposed Time	Temperature (°C) *	Color	Peak Area	% Degradation
None	None	None	Clear	29894.4	----
0.01 N HCl	24 hours	75	Clear	26868.3	8.80
0.01 N NaOH	One hour	75	Clear	27074.50	8.10
0.05 % H_2O_2	24 hours	75	Clear	28837.0	6.50
Mixture solution	-	-	Clear	27144.12	9.20

*: Heat it on a heating block at 75 °C for the specified duration.

Table S2. Method Robustness Results Variations for Mixed Degradation Sample

Parameters	Conditions	Retention time (min.)	Tailing Factor	Peak Area (mAU)	Number of Theoretical plates
pH of buffer	2.7	11.3	1.05	30365	12566
	2.9	12.2	1.04	31341	14771
	3.1	11.95	1.05	30855	14135
Flow rate mL/min	0.8	14.16	1.04	35135	16018
	1	12.2	1.05	31341	14771
	1.2	10.2	1.05	25578	13922
Wavelength	247	12.29	1.05	30627	14959
	249	12.2	1.05	31341	14771
	251	12.08	1.06	31187	14433
% B composition	25	17.67	0.98	29665.0	16990
	30	12.2	1.05	31341	14771
	35	8.23	1.13	27703	10774
Injection Volume (μL)	13	12.2	1.06	27899	15601
	15	12.2	1.05	31341	14771
	17	12.38	1.03	34776	13973

Table S3. Limit of Detection Study Results

Sample #	Concentration (ppm)	Peak Area	Retention time	Signal to Noise ratio
1	10.0	415.81	11.54	117.2
2	5.0	185.54	11.90	41.0
3	2.0	107.47	11.47	31.7
4	1.5	69.40	11.49	18.1
5	1.3	60.13	11.62	16.2
6	1.0	51.91	11.79	10.80
7	0.90	40.63	12.0	7.50
8	0.50	25.17	11.75	6.20
9	0.40	17.58	11.76	5.70

10	0.30	12.85	11.85	4.0
11	0.20	9.91	11.83	2.6

Table S4. Limit of Quantitation Study Results

Sample #	Concentration (ppm)	Peak Area	Retention time	Signal to Noise ratio
1	1.0	50.44	11.58	11.8
2	1.0	51.55	11.68	11.0
3	1.0	52.44	11.70	11.7
4	1.0	49.97	11.70	10.7
5	1.0	52.18	11.68	12.1
6	1.0	52.03	11.63	11.2
7	1.0	51.56	11.58	12.2
8	1.0	51.16	11.50	10.6
9	1.0	52.13	11.53	10.8
10	1.0	51.73	11.55	10.5
Average		51.52		11.26
Standard Deviation		0.793		0.64
%RSD		1.54		5.68
